# Therapeutic Potential of 4-Hexylresorcinol in Preserving Testicular Function in Streptozotocin-Induced Diabetic Rats

**DOI:** 10.3390/ijms25084316

**Published:** 2024-04-13

**Authors:** Ji-Hyeon Oh, Je-Yong Choi, Dae-Won Kim, Seong-Gon Kim, Umberto Garagiola

**Affiliations:** 1 Department of Oral and Maxillofacial Surgery, College of Dentistry, Gangneung-Wonju National University, Gangneung 25457, Republic of Korea; oms@gwnu.ac.kr; 2 Department of Biochemistry and Cell Biology, Cell and Matrix Research Institute, School of Medicine, Kyungpook National University, Daegu 41944, Republic of Korea; 3 Department of Oral Biochemistry, College of Dentistry, Gangneung-Wonju National University, Gangneung 25457, Republic of Korea; kimdw@gwnu.ac.kr; 4 Biomedical, Surgical and Oral Sciences Department, Maxillofacial and Dental Unit, School of Dentistry, University of Milan, 20122 Milan, Italy; umberto.garagiola@unimi.it

**Keywords:** 4-hexylresorcinol, diabetes, testis, testosterone, apoptosis

## Abstract

It is known that many diabetic patients experience testicular atrophy. This study sought to investigate the effect of 4-hexylresorcinol (4HR) on testicular function in rats with streptozotocin (STZ)-induced diabetes, focusing on testicular weight, sperm motility, histological alterations, and serum testosterone levels to understand the efficacy of 4HR on testes. Our findings reveal that 4HR treatment significantly improves testicular health in diabetic rats. Notably, the STZ group exhibited a testicular weight of 1.22 ± 0.48 g, whereas the STZ/4HR group showed a significantly enhanced weight of 1.91 ± 0.26 g (*p* < 0.001), aligning closely with the control group’s weight of 1.99 ± 0.17 g and the 4HR group’s weight of 2.05 ± 0.24 g, indicating no significant difference between control and 4HR groups (*p* > 0.05). Furthermore, the STZ/4HR group demonstrated significantly improved sperm motility compared to the STZ group, with apoptotic indicators notably reduced in the STZ/4HR group relative to the STZ group (*p* < 0.05). These results underscore the therapeutic potential of 4HR for maintaining testicular function under diabetic conditions.

## 1. Introduction

Diabetes mellitus (DM), a metabolic disorder characterized by chronic hyperglycemia, significantly affects various body systems, including the circulatory and neuroendocrine systems [1]. The male reproductive system is notably susceptible to complications associated with Type 1 diabetes mellitus (Type 1 DM) [2]. These complications include erectile dysfunction (ED) and subfertility, both of which are more prevalent in men with Type 1 DM compared to their non-diabetic counterparts [3]. ED, a common issue in this demographic, is characterized by the inability to achieve or maintain an erection adequate for sexual intercourse [4].

Type 1 DM has been found to contribute to significant alterations in male gonadal physiology in both experimental models and clinical cases [2]. According to meta-analyses conducted on studies focusing exclusively on men with type 1 DM, there is evidence supporting a detrimental effect of diabetes on sperm motility [5]. Type 1 DM individuals exhibit a significantly higher prevalence of immaturity- and apoptosis-related defects in spermatozoa compared to the control group, suggesting an association with impaired spermatogenesis that could potentially impact male fertility [6]. In addition, type 1 DM significantly disrupts the hypothalamic–pituitary–testicular axis, which is vital for regulating male reproductive functions [6]. This disruption is partly due to the central role of insulin in modulating pituitary and gonadal activities. Insulin is known to enhance luteinizing hormone-releasing hormone (LHRH)-induced gonadotrophin secretion, a critical process for reproductive function, as demonstrated in vitro [7]. In type 1 DM, where insulin deficiency is prevalent, there is reduced glucose utilization by anterior pituitary cells, leading to impaired pituitary functions [8]. Consequently, this insulin deficiency has been associated with a decreased response of follicle-stimulating hormone (FSH) and luteinizing hormone (LH) to gonadotropin-releasing hormone (GnRH) administration, as observed in insulin-deficient rats [9]. These alterations in hormonal signaling and response contribute to impaired testicular function, affecting sperm production and quality, and highlight the complex and multifaceted impact of type 1 DM on male fertility at the molecular level [6]. 

In type 1 DM, overactivation of metabolic pathways such as the polyol pathway, protein glycosylation, and glucose auto-oxidation lead to increased reactive oxygen species (ROS) production [10,11]. This exacerbates cellular oxidative stress [12], diminishing antioxidant capacity in testicular mitochondria [13]. Enhanced oxidative stress in type 1 DM is linked to germ cell apoptosis and DNA damage, impairing sperm function and fertility [14]. Studies show that oxidative imbalance in diabetes, particularly affecting sperm membranes rich in polyunsaturated fatty acids, is a principal pathophysiological state in the testis, with antioxidative treatments shown to mitigate damage [15].

Various strategies have been proposed to counteract these detrimental effects. Metformin, although typically used in type 2 DM, has shown potential in reducing testicular apoptosis in type 1 DM models [16]. Other compounds, such as icariin and loganin, combined with the nuclear factor kappa-light-chain-enhancer of activated B cells (NF-κB) inhibitors, have shown promise in protecting against type 1 DM-induced testicular damage [17,18]. Furthermore, the administration of antioxidants may enhance protection against diabetes-induced impairment by decreasing apoptosis in testicular tissue [19,20].

4-Hexylresorcinol (4HR), a phenolic compound derived from plants, exhibits potent antioxidant activity [21]. In rat models of DM, 4HR has shown effectiveness in promoting capillary regeneration and improving wound healing [22]. Additionally, the administration of 4HR has been observed to lower blood sugar levels [23]. Given that both types of DM are characterized by chronic inflammation and disturbances in the immune system, inhibiting the nuclear factor kappa-light-chain-enhancer of the activated B cells (NF-κB) signaling pathway could mitigate some of the foundational processes of the disease. Since 4HR has been shown to inhibit NF-κB [24,25], its administration may offer a therapeutic strategy to alleviate the disturbances induced by DM. The administration of 4HR, through its roles in enhancing wound healing [22], exerting antioxidant effects [21], inhibiting the NF-κB pathway [24,25], and reducing serum glucose levels [23], may prove advantageous in mitigating complications in the testes induced by STZ-induced DM. While the administration of 4HR has been shown to lower serum testosterone levels and reduce mandibular size in growing healthy rats, indicating complex effects on testosterone dynamics [26], the implications of these findings for its therapeutic use, especially in diabetic testes, remain uncertain and warrant further investigation.

Our study aims to investigate the protective effects of 4HR on the male reproductive system using the streptozotocin (STZ)-induced DM model in rats, specifically focusing on its potential to alleviate apoptosis in the testicular tissue. Previous research indicates that in diabetic rats induced by STZ, the testicles exhibit a higher level of apoptosis, suggesting significant stress on the reproductive system [14]. To evaluate whether 4HR administration can alleviate this apoptotic stress, we aim to assess several key indicators of testicular health and function. We specifically focus on evaluating the size of the testicles and sperm motility, as these are direct measures of reproductive health. Additionally, we conduct histological examinations to observe any cellular or structural changes within the testicular tissue. To understand the underlying biological mechanisms, we measure serum testosterone levels, which play a critical role in male reproductive function. Furthermore, we examine the expression of apoptosis-associated proteins through both immunohistochemistry and Western blot analysis, providing comprehensive insights into the cellular response to 4HR treatment. By assessing these parameters, our study aims to elucidate the potential benefits of 4HR on the male reproductive system in the context of diabetes-induced testicular stress.

This study reveals the innovative therapeutic potential of 4HR in ameliorating diabetic testicular dysfunction, a significant step forward in understanding and treating this complication. Through a comprehensive evaluation encompassing testicular size, sperm motility, histological alterations, and serum testosterone levels, our findings demonstrate that 4HR treatment substantially improves testicular health in a rat model of STZ-induced diabetes. Notably, the enhancements in testicular size and sperm motility, alongside a marked reduction in apoptotic indicators in the STZ/4HR group compared to the STZ group, underscore 4HR’s beneficial impact.

## 2. Results

### 2.1. Effect of STZ and 4HR on Testis Weight and Sperm Motility in Diabetic and Non-Diabetic Rats

**Figure 1:**
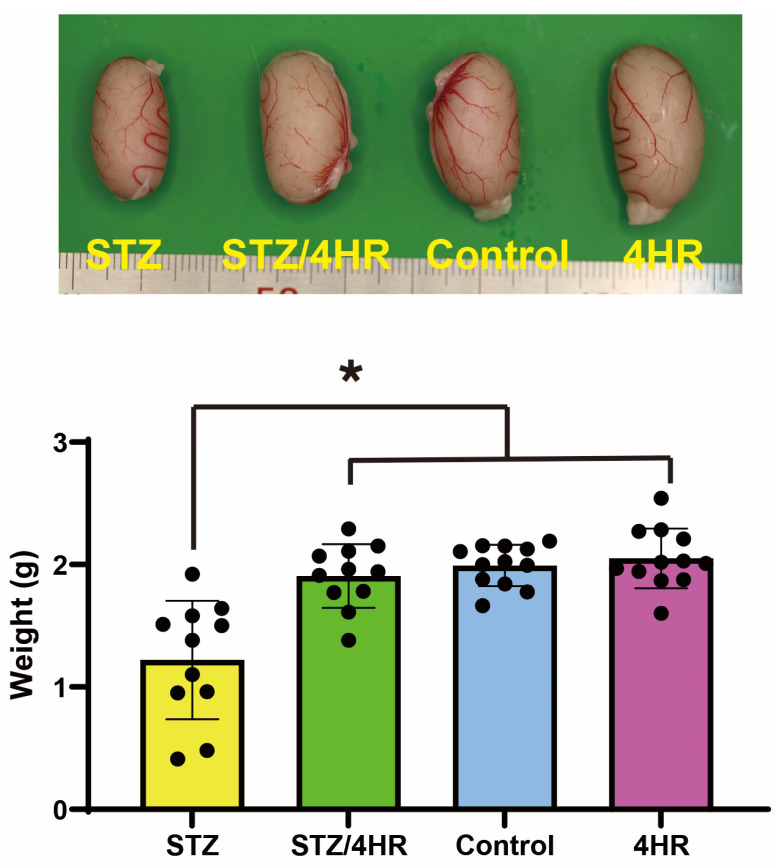
The weight of testis in each group. When comparing the STZ group to the other groups, the difference was statistically significant (* *p* < 0.05).

The STZ group exhibited a testicular weight of 1.22 ± 0.48 g (mean ± standard deviation), while the STZ/4HR group’s weight was 1.91 ± 0.26 g (Figure 1), revealing a statistically significant difference between these two groups (*p* < 0.001). The control group’s testicular weight was 1.99 ± 0.17 g, and the 4HR group’s weight was 2.05 ± 0.24 g. No significant difference was found between the control and 4HR groups (*p* > 0.05). When comparing the STZ/4HR group to either the control or 4HR group, the difference was not statistically significant. However, the STZ group’s testicular weight was significantly lower than both the control and 4HR groups (*p* < 0.001).

**Figure 2:**
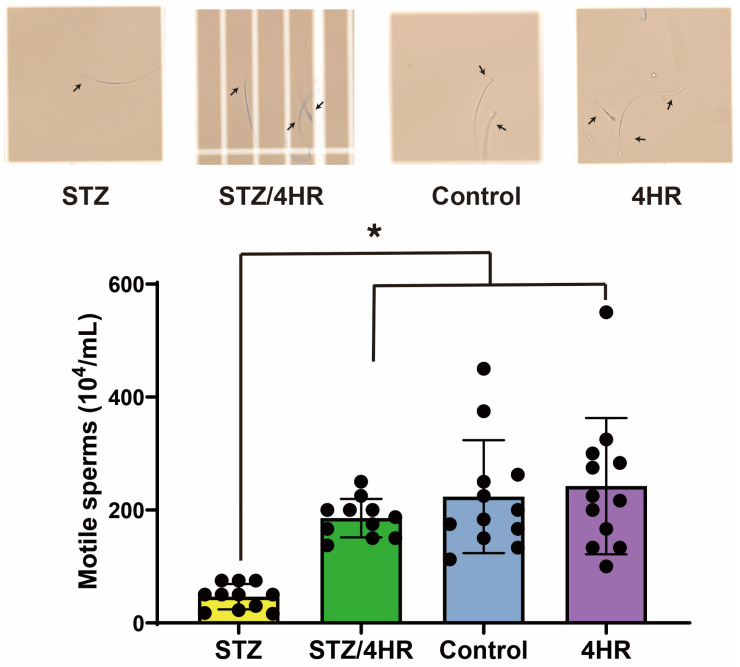
The sperm motility assay. In the sperm motility assay, a statistically significant difference in sperm mobility was observed between the STZ group and the other groups (* *p* < 0.05). Specifically, the STZ group demonstrated fewer mobile sperm (arrows), as evidenced in Video S1.

In the sperm motility assay, the STZ-treated group exhibited a significant reduction in the number of motile sperm compared to the non-diabetic rats in the control and 4HR groups. Specifically, the STZ group had a motile sperm count of 46.5 ± 22.6 × 10^4^/mL. In contrast, the STZ/4HR treatment group showed a substantial increase in motile sperm count to 185.6 ± 34.1 × 10^4^/mL, as depicted in Figure 2, highlighting a statistically significant improvement (*p* = 0.002). The control group had a motile sperm count of 223.6 ± 100.0 × 10^4^/mL, while the 4HR group presented a count of 242.4 ± 120.6 × 10^4^/mL. There was no significant difference in sperm motility between the control and 4HR groups (*p* > 0.05), indicating that 4HR treatment did not adversely affect sperm motility. Furthermore, when comparing the STZ/4HR group to either the control or 4HR group, no significant differences were found. Notably, the number of motile sperm in the STZ group was significantly lower than in both the control and 4HR groups (*p* < 0.001), underscoring the negative impact of STZ on sperm motility that was mitigated by 4HR treatment.

### 2.2. Effects of 4HR on Sperm Morphology, Apoptosis, and Serum Testosterone Levels in Diabetic Rats

**Figure 3:**
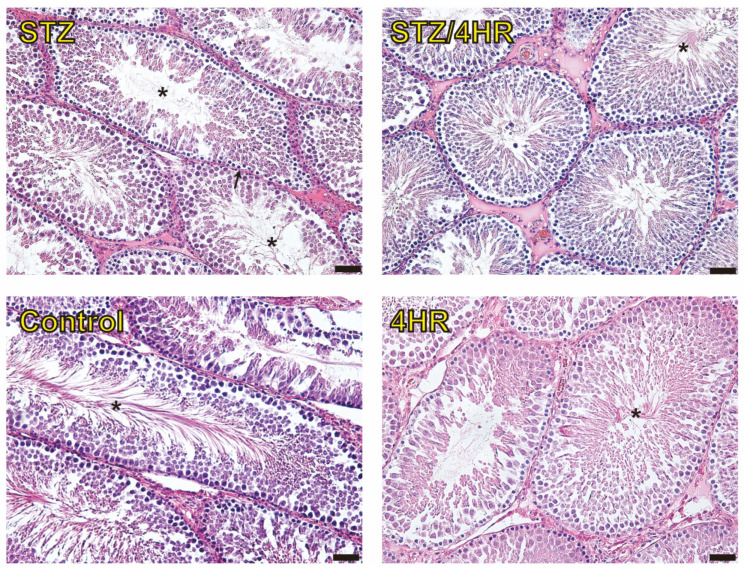
Histological images. In the STZ group, abnormal sperm morphology was observed, characterized by sparsely distributed flagella (denoted by *) and a decreased sperm density within the seminiferous tubules (arrow). Additionally, the basement membranes of the seminiferous tubules were often thickened. Conversely, the STZ/4HR group displayed an increased flagella density, aligning closely with both the control and 4HR groups, with no significant differences noted among them (bar = 50 μm).

Under hematoxylin and eosin (HE) staining, the STZ group exhibited abnormal sperm morphology, including a poorly developed tail portion, and a higher count of immature sperm cells (Figure 3). In contrast, the STZ/4HR group showed a greater proportion of mature sperm cells with well-developed tails. There was no prominent morphological difference observed when comparing the STZ/4HR group to either the control or 4HR group.

**Figure 4:**
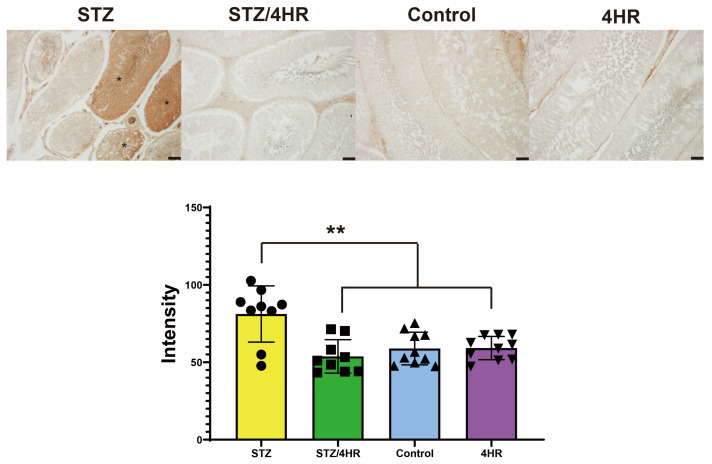
Immunostaining for cleaved caspase-3 (c-caspase-3) demonstrated a statistically significant difference in immunoreactivity intensity across the groups (** *p* < 0.001). Detailed analysis indicated a pronounced elevation in c-caspase-3 expression within the STZ group compared to the others, with the STZ group showcasing seminiferous tubules that were highly positive (*). Significantly, *p*-values of <0.001 for the STZ/4HR group and 0.002 for both the control and the 4HR group were observed, underscoring these differences (bar = 100 μm).

**Figure 5:**
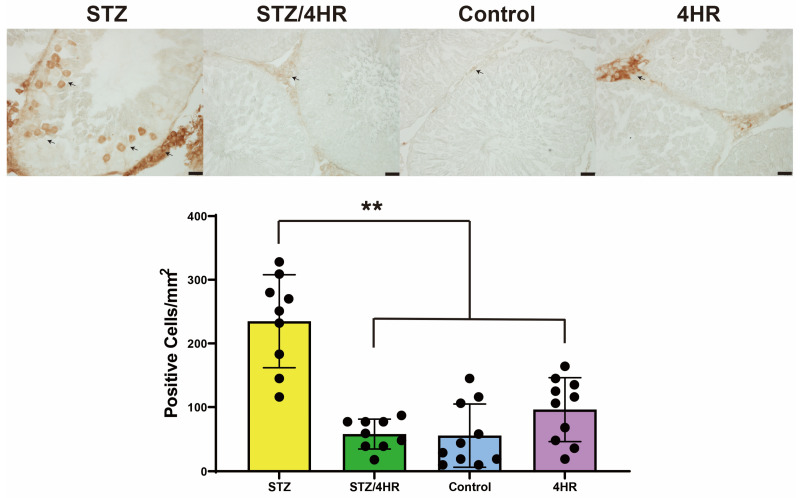
Immunostaining for p53. The figure depicts a high concentration of p53-positive cells in the STZ group, indicated by arrows. A comparison across groups reveals a significantly greater number of p53-positive cells in the STZ group relative to the other groups (** *p* < 0.001). The specimen was counterstained using methyl green, and the scale bar represents 20 μm.

The intensity of cleaved-caspase-3 (c-caspase-3) was significantly increased in the STZ group compared to the other groups, with *p*-values of <0.001 for the STZ/4HR group and 0.002 for the control and the 4HR group (Figure 4). In p53 immunostaining, the STZ-treated group exhibited a significant increase in the number of p53-positive cells compared to the non-diabetic rats in the control and 4HR groups. Specifically, the STZ group had a p53 positive cell count of 234.9 ± 73.0/mm^2^. In contrast, the STZ/4HR treatment group showed a substantial decrease in p53-positive cell count to 57.9 ± 23.3/mm^2^, as depicted in Figure 5, highlighting a statistically significant difference (*p* < 0.001). The control group had a p53-positive cell count of 55.6 ± 49.3/mm^2^, while the 4HR group presented a count of 96.2 ± 50.0/mm^2^. There was no significant difference in sperm motility between the control and 4HR groups (*p* > 0.05), indicating that 4HR treatment did not increase the number of p53-positive cells.

**Figure 6:**
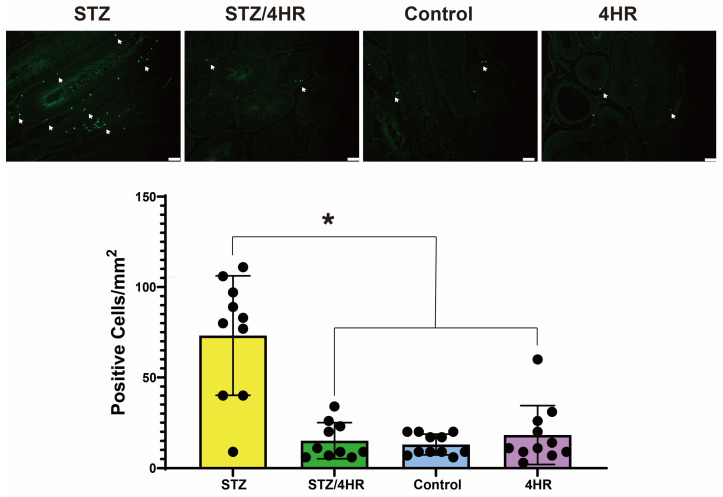
The quantification of TUNEL-positive cells per square millimeter. The STZ group exhibited a considerably higher count of TUNEL-positive cells (arrows) in comparison to the other groups (* *p* < 0.001) (bar = 100 μm).

In addition, the terminal deoxynucleotidyl transferase dUTP nick end labeling (TUNEL)-positive cells per square millimeter were counted (Figure 6). A comparative analysis across groups revealed that the STZ group exhibited a significantly higher number of TUNEL-positive cells compared to the other groups. The difference was statistically significant (*p* < 0.001).

**Figure 7:**
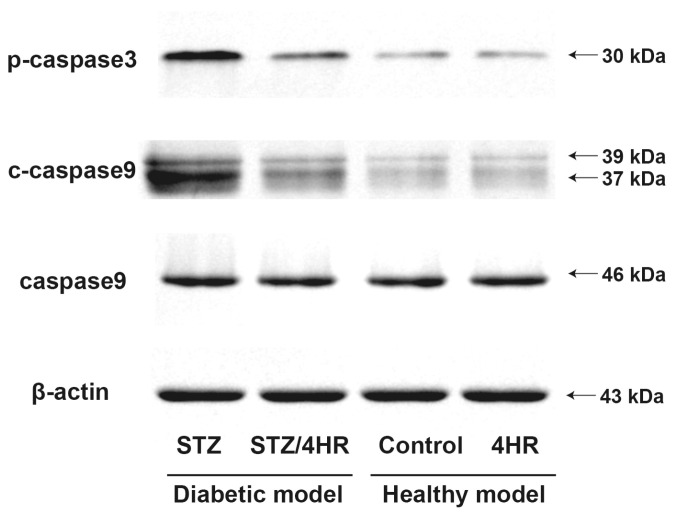
The Western blot for selected apoptosis marker. Fresh testes acquired from each group were used for protein extraction. The expression levels of p-caspase3 and c-caspase9 were higher in the STZ group than in other groups.

Fresh samples of testes were used for the analysis of western blot. The results of the Western blot were in accord with the immunohistochemical staining. The expression levels of p-caspase3 and c-caspase9 were higher in the STZ group than in other groups (Figure 7).

**Figure 8:**
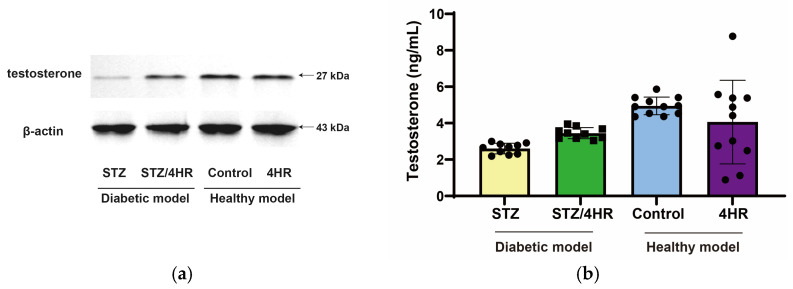
The testosterone level in the testes and the serum. (**a**) The testosterone-bound proteins were detected by Western blot in the testes. The level of testosterone was lower in the STZ group compared to the other groups (**b**) The serum testosterone level. STZ group showed significantly lower levels of serum testosterone compared to those of healthy models (*p* < 0.05).

When serum testosterone level was measured, it was 2.61 ± 0.29 ng/mL for the STZ group and 3.45 ± 0.31 ng/mL for the STZ/4HR group (Figure 8). When comparing the STZ group and STZ/4HR group with independent samples t-test, the difference between groups was statistically significant (*p* < 0.001). However, there was no significant difference in the post hoc ANOVA test (*p* > 0.05). When comparing the STZ group to the healthy model, the difference between groups was statistically significant (*p* < 0.05).

## 3. Discussion

In this study, 4HR administration decreased the expression level of apoptosis-associated proteins (Figure 7) and the intensity of c-caspase-3 immunoreactivity (Figure 4). Consequently, testicular atrophy and diabetes-induced decreased sperm motility were mitigated by 4HR administration (Figure 1 and Figure 2). Complementarily, sitagliptin, an anti-diabetic medication, has been reported to regulate blood glucose levels and reduce apoptosis and endoplasmic reticulum stress in diabetic male rats, suggesting its potential in preserving testicular tissue integrity [27]. Similarly, metformin, another diabetes treatment, is not only known for its glycemic control improvements but also for counteracting diabetes-induced testicular oxidative stress, inflammation, and apoptosis, thereby potentially enhancing male fertility in diabetic states [28]. Moreover, metformin has been shown to have protective effects against testicular damage in both diabetes and prostate cancer models, highlighting its role in reducing oxidative stress and apoptosis while enhancing antioxidant capacity [29]. These studies collectively underscore the importance of investigating diabetes medications like 4HR, sitagliptin, and metformin for their additional benefits in protecting against histopathological damage in testicular tissues, potentially improving reproductive health outcomes in diabetic individuals.

Several earlier studies have suggested potential interventions to guard against type 1 diabetes-induced testicular atrophy, including specific medications, antioxidant-rich diets, and dietary supplements [30,31]. Our findings support these previous studies and suggest that the antioxidant activity of 4HR may mediate its protective effects on testicular function in the context of DM [19]. In addition to the antioxidant activity of 4HR, we observed a significant increase in leucine-rich repeat-containing G-protein-coupled receptor 4 (LGR4) expression (Supplementary Figure S1), a protein known to protect cells against oxidative damage for testicular function [32]. The upregulation of LGR4 by 4HR might be helpful in testicular protection alongside its antioxidant effect [33].

Recent advancements in the field further underscore the significance of our findings. Elshafey et al. [34] demonstrated the protective effect of stevia on diabetic-induced testicular damage, emphasizing the potential of natural compounds in ameliorating diabetes-related reproductive issues. This aligns with our observations of 4HR’s efficacy. Additionally, Zheng et al. [35] explored the protective effects of chromium picolinate against testicular damage in STZ-induced type 1 diabetic rats, emphasizing its role in anti-inflammation, anti-oxidation, apoptosis inhibition, and regulation of the transforming growth factor-β1 (TGF-β1)/Smad pathway. Similarly, Lu et al. [36] demonstrated the protective effects of icariin against testicular dysfunction in type 1 diabetic mice via adenosine monophosphate-activated protein kinase (AMPK)-mediated nuclear factor erythroid 2–related factor 2 (Nrf2) activation and NF-κB p65 inhibition. These studies, along with others exploring the efficacy of natural compounds like stevia and their molecular mechanisms, highlight a growing interest in natural therapeutics for diabetic complications. Our research contributes to this evolving discourse, suggesting 4HR as another promising candidate for mitigating diabetic testicular dysfunction. The convergence of these findings underscores the potential of multifaceted approaches in addressing the complex interplay between diabetes and reproductive health. 

Interestingly, the administration of 4HR significantly decreased the count of apoptotic cells within the testis, typically heightened in diabetic conditions (Figure 6). This aligns with other research, including an initial investigation into the effects of 4HR on circumvallate papillae taste buds in diabetic and healthy rats, which found the compound effective in mitigating taste bud apoptosis, especially in DM-induced taste issues [33]. Similarly, the administration of metformin [37] or insulin [38] in type 1 DM decreased the count of p53-positive cells within the testis. Our study suggests that 4HR may alleviate the increase in apoptotic cells within the testis induced by diabetes, further protecting testicular function and potentially mitigating other diabetes-related complications.

Our study’s findings in STZ-induced diabetic animal models, especially regarding serum testosterone levels (Figure 7), present an intriguing contrast to established trends. Typically, such models exhibit decreased serum testosterone levels due to impaired hypothalamic–pituitary–gonadal (HPG) axis function and direct testicular damage [39]. However, our STZ/4HR group showed a significant increase in testosterone levels (Figure 7). This deviation suggests potential protective or compensatory mechanisms at play. Normally, the HPG axis, disrupted in diabetes, plays a crucial role in testosterone regulation [39]. In type 2 DM, factors like hyperglycemia and insulin resistance detrimentally affect this axis, leading to lower testosterone production [40]. Our results indicating elevated testosterone levels in diabetic rats, contrary to typical hypogonadism observed in STZ-induced diabetic animal models, raise questions about the unique impacts of 4HR on the HPG axis and testosterone synthesis. 

Additionally, diabetes-associated oxidative stress and inflammatory responses are known to cause indirect testicular damage, further impacting testosterone synthesis [34]. The introduction of 4HR in our STZ-induced model appears to have mitigated these effects. 4HR is known for its antioxidant properties [19], which might have contributed to protecting testicular cells from oxidative stress and inflammation typically associated with diabetes. This protective effect could explain the preservation or enhancement of testosterone production, despite the diabetic condition. Moreover, our results hint at a potential shift in mitochondrial metabolism between diabetic and non-diabetic conditions, as indicated by the altered testosterone levels. Mitochondria play a crucial role in steroidogenesis, and their dysfunction is often implicated in the pathophysiology of diabetes [41]. It is plausible that 4HR may influence mitochondrial function [42], thereby altering the metabolic pathways involved in testosterone synthesis. This aspect, however, warrants further investigation.

Despite the promising results of our study, several limitations must be acknowledged, and important considerations for future research directions are here proposed. First, our study utilized a rat model, which may not entirely represent the complex pathophysiology of diabetes and testicular dysfunction in humans. Thus, future studies should consider replicating these experiments in more complex animal models or human tissues to enhance the translatability of our findings. Furthermore, while our research provides insights into the potential benefits of 4HR, it is crucial to also consider the possible health risks associated with its use. 4HR has been indicated to have potential adverse effects, which need to be thoroughly understood and addressed [22,43]. Future research should aim to identify the safe dosage thresholds and long-term effects of 4HR, considering different demographics and comorbidities. In our study, the effects of a single dosage of 4HR over an 8-week treatment period were examined. Future studies should not only explore varying dosages and treatment durations but also different administration routes. This would help in ascertaining the most effective and safest therapeutic strategy for 4HR use. Additionally, while we have hypothesized the protective effects of 4HR as being due to its antioxidant activity, the precise biochemical and molecular mechanisms remain to be fully elucidated. Detailed studies focusing on these pathways are critical for understanding the therapeutic potential and safety profile of 4HR. Moreover, considering the potential health risks of 4HR, it is imperative to explore protective strategies or alternative compounds that could offer similar therapeutic benefits with reduced risk. Research into natural antioxidants, molecular analogs of 4HR, or combination therapies could provide safer and more effective options for managing diabetic-induced testicular dysfunction.

## 4. Materials and Methods

### 4.1. Animal Study

The animal study received approval from the Institutional Animal Care and Use Committee at Gangneung-Wonju National University (GWNU-2021-22 and was approved on 23 November 2021). Sprague Dawley outbred rats were purchased from Samtako (Osan, Republic of Korea) for this experiment. The rats consumed, on average, 10 g of food and 12 mL of water per day for each 100 g of body weight. Correspondingly, the food efficiency ratio was determined to be approximately 1.7. A total of 20 male rats, 7 weeks old, were utilized. When the rats received STZ injection, their body weight ranged from 270 to 290 g. Rats fasted for 6–8 h before STZ injection but had access to water ad libitum. To induce diabetes, a 40 mg/kg STZ injection was administered intravenously via the tail vein [44,45]. Rats were then placed back in their cages and given standard food and 10% sucrose water. Three days after the STZ injection, blood glucose levels at fasting state were measured from the tail vein using a monitoring system. Rats with blood glucose levels above 300 mg/dL were chosen for further investigation. Rats with inadequate blood glucose levels received another STZ injection. Furthermore, a postmortem examination of the pancreas was performed to confirm the expected pathological changes associated with Type 1 DM. These examinations revealed significant beta-cell destruction, consistent with Type 1 DM pathology, and have been included as Supplementary Data. Additional confirmation was conducted via postmortem examination of the pancreas (Supplementary Figure S2). STZ-injected rats were randomly assigned to either the untreated control group (STZ group) or the 4HR injected group (STZ/4HR group). After verifying the diabetic status, 4HR was administered subcutaneously at a dosage of 10 mg/kg weekly for the 8-week duration of the study. In our previous study, 1.28 or 128 mg/kg/week of 4HR was given to accelerate orthodontic tooth movement [46]. Though the therapeutic effect was dosage-dependent, the gap was not big. In a subsequent study, 12.8 mg/kg/week of 4HR was enough to inhibit histone deacetylase activity [47]. For the simple calculation of dosage, 10 mg/kg/week was used for this study. Injection volume was adjusted between 0.1 and 0.2 cc. One animal from each group was dead. At 16 weeks of age, the rats (n = 9 for each group) were humanely euthanized. The animals were anesthetized using enflurane and given an additional injection, a combination of Zoletil 50 (15 mg/kg; Vibac, Carros, France) and Rumpun (0.2 mL/kg; Bayer Korea, Seoul, Republic of Korea). Blood samples were obtained from the heart and stored in tubes. After centrifugation at 1200 rpm for 5 min using a low-speed centrifuge (Model 416, Gyrozen, Gimpo, Republic of Korea), the serum supernatant was collected for subsequent analysis. Following blood collection, the animals were euthanized with a 4% paraformaldehyde injection. Testes were removed and weighed. Subsequently, the tissue samples were divided into two groups. One group was designated for paraffin embedding, while the other was utilized for protein extraction and Western blot analysis.

For this study, an additional 40 testes were obtained from experiments on STZ-not-injected rats (GWNU-2021-21 and was approved on 23 November 2021) that were of the same age as the rats used in this experiment. Out of these 20 testes, 10 were assigned to the untreated control group, while the other 10 received the same dosage of 4HR as used in this experiment and were assigned to the 4HR group. These testes were used to measure their weight and for histological analysis. Additionally, supplementary experiments were conducted for 8 rats (GWNU-2023-26 and was approved on 17 November 2023). Two additional rats were added to each group. Therefore, the STZ and STZ/4HR groups had 11 rats each, and the Control and 4HR groups had 12 rats each. 

### 4.2. Sperm Motility Assay

The motility assay of sperm from the cauda epididymis was conducted immediately following euthanasia [48]. The caudal part was excised and placed into a conical tube filled with Hank’s balanced salt solution (HBSS), supplemented with calcium and magnesium, to support sperm viability. In a clean bench environment, the cauda epididymis was finely chopped to facilitate the release of sperm. This setup allowed motile sperm to “swim out” from the epididymal tissue into the HBSS medium (5 mL) over 30 min at 37 °C, enabling their separation.

Subsequently, the suspension was centrifuged at 1000 rpm for 5 min to sediment the sperm. The supernatant was then carefully aspirated without disturbing the pellet to ensure the integrity of the collected sperm. To facilitate counting, the supernatant containing the motile sperm was diluted in a 1:10 ratio with HBSS.

A pipette was used to transfer the diluted sperm sample into the hemocytometer chamber. The motile sperm within the sample were quantified using a Neubauer chamber under a microscope. This procedure allowed for the accurate determination of motile sperm concentration, which was expressed as the number of motile sperm per milliliter (sperms/mL).

### 4.3. Histological Staining and Immunohistochemistry

For the hematoxylin and eosin (HE) staining, 5 μm sections of formalin-fixed paraffin-embedded tissue samples are deparaffinized by immersing them in xylene to remove the paraffin wax. Once deparaffinized, the tissue sections are rehydrated. This involves passing them through a graded series of alcohol solutions of decreasing concentrations—100%, 99%, and 95%. The sections are immersed in a solution of hematoxylin for 6 min, after which the sections are washed by running tap water for 20 min. The sections are then immersed in an acid–alcohol solution for seconds, which removes excess hematoxylin and differentiates the nuclei. The sections are then counterstained with eosin for 2 min. After the dehydration procedure, the section was mounted with a coverslip and mounting medium (Eukitt^®^ quick hardening mounting medium, CAT#: 03989, Sigma-Aldrich, St. Louis, MO, USA).

For the immunohistochemical staining, primary antibodies for testosterone (CAT#: sc-52242), p53 (CAT#: sc-126), and caspase-3 cleaved (c-caspase-3; CAT#: sc-22171-R) were purchased from Santa Cruz Biotechnology (Santa Cruz, CA, USA). The hydration procedure was in accord with HE staining. After blocking endogenous peroxidase activity by the application of 30% H_2_O_2_ (Samchun, Pyeongtaek, Republic of Korea) for 7 min, the slide was washed twice with phosphate-buffered saline (PBS). Then, blocking with protein block (CAT#: X0909, Agilent Technologies, Santa Clara, CA, USA) was undertaken for 1 h. Primary antibodies (dilution ratio 1:100) were applied to each section. Then, the section was incubated at 4 °C overnight. Then, the slide was washed 3 times with PBS, and a ready-made horse reddish peroxidase-conjugated secondary antibody (Real EnVision™, Dako, Glostrup, Denmark) was applied to the section for 30 min. Then, the slide was washed 3 times with PBS, and chromogen (DAB+ chromogen, Dako) was applied for 5 min. After the washing procedure with distilled water, a cover slip was applied with a mounting medium (Ultramount Aqueous Permanent Mounting Medium, Dako). 

The quantification of immunostaining was performed using Sigma Scan Pro version 5.0 (SPSS Inc., Chicago, IL, USA). The expression levels of cleaved caspase-3 were assessed specifically within this tissue. For each sample, regions of interest (ROIs) within the interstitial tissue were systematically selected across a predetermined number of microscopic fields. Specifically, 3 randomly selected fields per sample were analyzed to ensure representative and comprehensive data collection. The mean intensity of the staining in these selected ROIs was then quantified using a scale ranging from 0 (indicating no staining) to 255 (indicating maximum staining). For the quantification of p53 positive cells, the counts were standardized per square millimeter to facilitate comparison among different samples. In total, 46 samples were analyzed, providing a robust dataset for statistical analysis and comparison among the groups.

### 4.4. TUNEL Assay

The process of terminal deoxynucleotidyl transferase dUTP nick end labeling (TUNEL) staining was carried out using the In Situ Cell Death Detection kit from Roche, Basel, Switzerland. Initially, paraffin was removed from the tissue slide. Subsequently, trypsin in 0.01 N HCl was applied to the slide, which was then placed in humidified chambers at 37 °C for 30 min. The slide was then washed twice with phosphate-buffered saline with tween20 (PBST) for 5 min each time. Following this, the TUNEL reaction mixture was applied to the slides, which were then covered with a plastic slip. The reaction was allowed to proceed in a humidified dark chamber at 37 °C for 60 min. The slides were washed with PBST three times, each lasting 5 min. After the application of a mounting medium for fluorescence, the slides were examined under a fluorescent microscope. Nuclei that stained positive appeared as an intense green color. Specifically, 3 randomly selected fields per sample were analyzed to ensure representative and comprehensive data collection. The count of TUNEL-positive cells in the testis was determined for each square millimeter and the differences among the groups were compared.

### 4.5. Western Blot

Tissue samples were homogenized in a lysis buffer containing protease inhibitors. The lysate was centrifuged at 10,000× *g* for 10 min at 4 °C to remove cellular debris. The protein concentration of the supernatant was measured using the Bradford method [29]. The protein samples were mixed with SDS sample buffer and heated at 95–100 °C for 5 min. The equal concentration of protein samples was loaded onto an SDS-PAGE gel and we ran the gel using a Tris-glycine buffer. The separated proteins were transferred from the gel onto a PVDF membrane using a transfer apparatus. After the blocking procedure, the membrane incubated the membrane with antibodies against protein-bound testosterone (CAT#: sc-52242), caspase-3-phospho-s150 (CAT#: ab59425), caspase-9 (CAT#: sc-17784), and caspase-9 cleaved (c-caspase-9; CAT#: sc-22182) (dilution ratio = 1:1000) in the blocking buffer overnight at 4 °C. After the washing procedure, the membrane was incubated with a secondary antibody conjugated to a detection enzyme for 1 h at room temperature. After washing, a chemiluminescent substrate was applied to the membrane, and the protein bands were visualized using a chemiluminescence imaging system. The experiments described herein were performed in triplicate.

### 4.6. Measuring Serum Testosterone Level

Serum samples were collected from each group. Measuring was conducted with the commercially available kit (mouse/rat testosterone ELISA Kit, CAT#: ab285350, Abcam, Cambridge, UK) (measuring range: 0.1–18 ng/mL). The specimens were diluted using the assay buffer included in the kit. By employing the provided testosterone standard (0 to 18 ng/mL), the standard curve was obtained. A total of 50 µL of each standard and specimen was placed into the corresponding wells of the microplate, which was coated with an antibody specific to testosterone. Additionally, 50 µL of the supplied testosterone-HRP conjugate was introduced to each well. The microplate was then sealed with the adhesive strip included in the kit and incubated for 2 h at room temperature on a shaker. Following the removal of the adhesive strip, the microplate was washed thrice with the kit’s wash buffer. Subsequently, the substrate was incubated for 15 min at room temperature in the absence of light, after which 50 µL of the stop solution was added to each well. The absorbance of every well was measured at 450 nm with a microplate reader. The testosterone concentrations of the specimens were determined using the standard curve, which was generated by plotting the absorbance values of the standards against their respective concentrations.

### 4.7. Statistical Analysis

Statistical analysis was performed using GraphPad Prism version 9 (GraphPad Software, Boston, MA, USA). Results are presented as mean ± standard deviation. For comparing the two groups, an independent samples t-test was employed. When comparing multiple groups, an analysis of variance was conducted. Tukey’s multiple comparison test was utilized as the post hoc test. A significance level of *p* < 0.05 was established for the analyses.

## 5. Conclusions

This study underscores the therapeutic potential of 4HR in mitigating the effects of diabetic testicular dysfunction, highlighting its capability to enhance testicular function and structure under diabetic conditions. Notably, our comprehensive evaluation, including measurements of testicular size, sperm motility, histological alterations, and serum testosterone levels, demonstrates that 4HR treatment significantly improves testicular health in rats with STZ-induced diabetes. The observed enhancements in testicular size and sperm motility, coupled with the notable reduction in apoptotic indicators in the STZ/4HR group compared to the STZ group, affirm the beneficial impact of 4HR. While these findings are promising, they also emphasize the need for further investigations to fully elucidate the underlying mechanisms of 4HR’s action and to assess its long-term safety and efficacy. Future research, encompassing both animal models and clinical trials in human patients, is essential to validate these preliminary results and explore the potential of 4HR for clinical applications in managing diabetic-induced testicular dysfunction.

## Data Availability

All data were shown in the paper and there was no additional dataset.

## Supplementary Materials

The following supporting information can be downloaded at https://www.mdpi.com/article/10.3390/ijms25084316/s1.
