# Supplementary material for: Therapeutic Potential of 4-Hexylresorcinol in Preserving Testicular Function in Streptozotocin-Induced Diabetic Rats
Source: Int J Mol Sci. 2024 Apr 13;25(8):4316. doi: 10.3390/ijms25084316 (PMC11050698; doi:10.3390/ijms25084316)

Figure S1. Immunohistochemical images. The expression level of LGR4 was lower in the STZ group than in the other groups (bar=100μm).

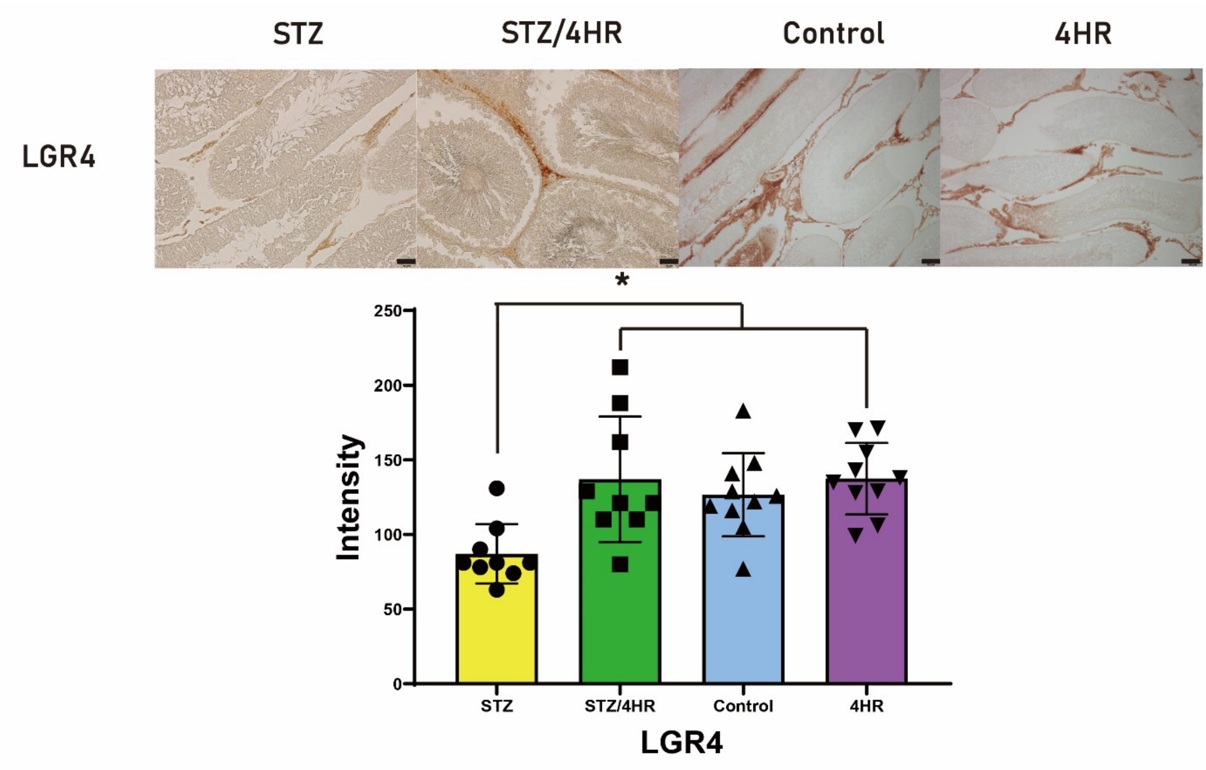

Figure S2. Histology and immunostaining of the pancreas (scale bar = 50μm) confirm the successful induction of type 1 diabetes through autopsy. The untreated group exhibited well-preserved islets of Langerhans (*), in contrast to the STZ-injected group, which demonstrated significant destruction of these islets (*). Correspondingly, insulin immunoreactivity was marked by strong intensity and extensive coverage in the untreated group (arrow), whereas the STZ-injected group displayed markedly reduced immunointensity and diminished areas of reactivity (arrow).

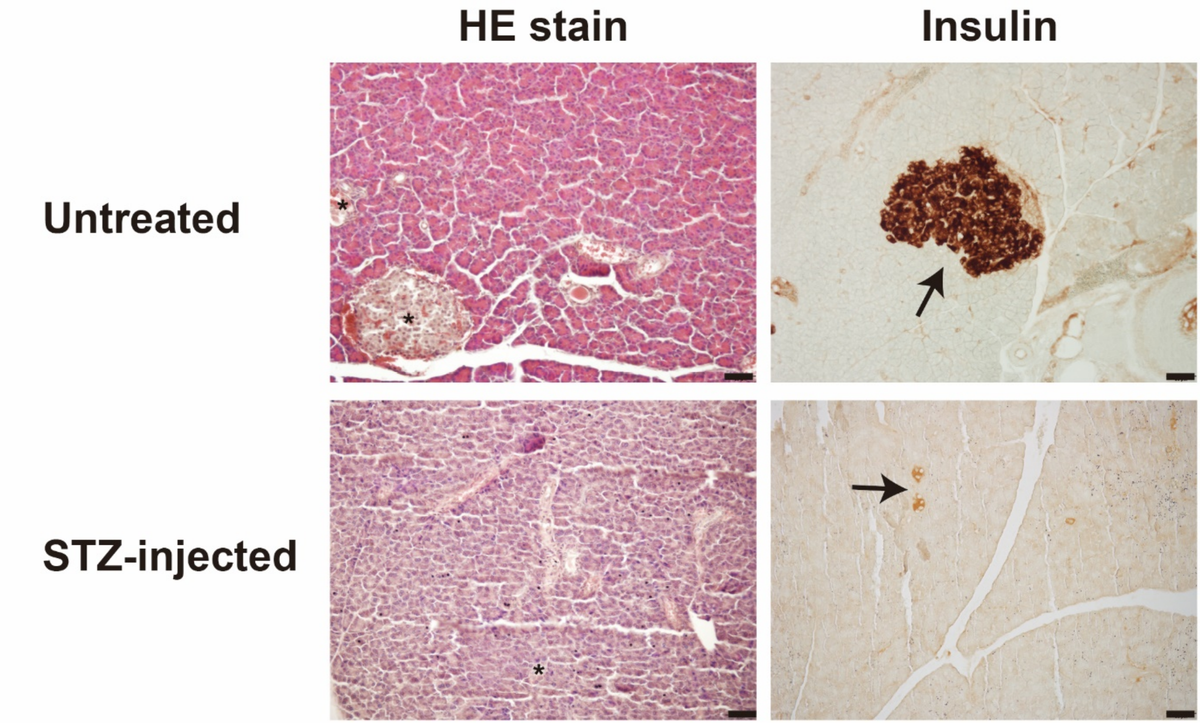

Full length blot image

The expression level of LGR4 was increased by 4HR administration.

A. β-actin (from left lane to right, **1:** rat1's testis from STZ group, **2:** rat2's testis from STZ group, **3:** rat3's testis from STZ group, **4:** rat4's testis from STZ group, **5:** rat5's testis from STZ group, **6:** rat1's testis from STZ/4HR group, **7:** rat2's testis from STZ/4HR group, **8:** rat3's testis from STZ/4HR group, **9:** rat4's testis from STZ/4HR group, **10:** rat5's testis from STZ/4HR group)

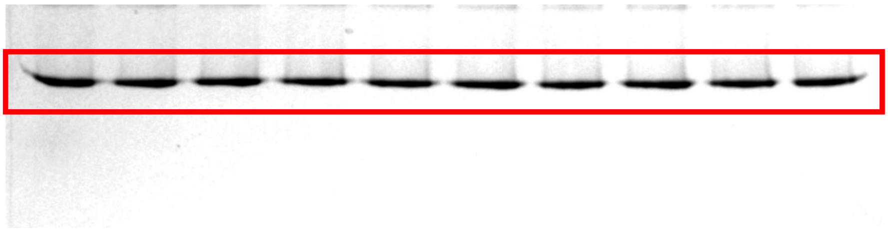

B. LGR4 (**1:** rat1's testis from STZ group, **2:** rat2's testis from STZ group, **3:** rat3's testis from STZ group, **4:** rat4's testis from STZ group, **5:** rat5's testis from STZ group, **6:** rat1's testis from STZ/4HR group, **7:** rat2's testis from STZ/4HR group, **8:** rat3's testis from STZ/4HR group, **9:** rat4's testis from STZ/4HR group, **10:** rat5's testis from STZ/4HR group)

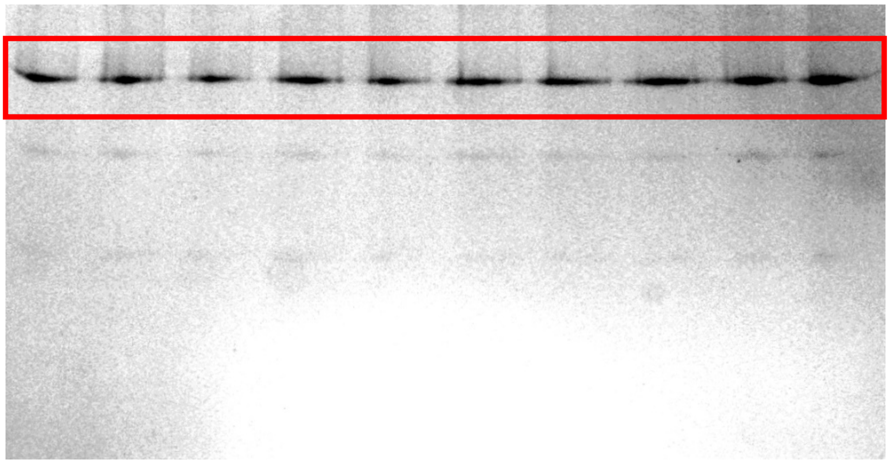

Supplement: Supplementary file 1 [file ijms-25-04316-s001.zip › ijms-2945441-supplementary.pdf]
